# Diabetes-Induced Dysfunction of Mitochondria and Stem Cells in Skeletal Muscle and the Nervous System

**DOI:** 10.3390/ijms18102147

**Published:** 2017-10-14

**Authors:** Shin Fujimaki, Tomoko Kuwabara

**Affiliations:** 1 Musculoskeletal Molecular Biology Research Group, Basic and Translational Research Center for Hard Tissue Disease, Graduate School of Biomedical Sciences, Nagasaki University, 1-7-1 Sakamoto, Nagasaki 852-8523, Japan; shin.fujimaki0526@gmail.com; 2 Biotechnology Research Institute for Drug Discovery, Department of Life Science and Biotechnology, National Institute of Advanced Industrial Science and Technology (AIST), Central 5, 1-1-1 Higashi, Tsukuba 305-8565, Ibaraki, Japan

**Keywords:** diabetes, mitochondria, satellite cells, neural stem cells, exercise

## Abstract

Diabetes mellitus is one of the most common metabolic diseases spread all over the world, which results in hyperglycemia caused by the breakdown of insulin secretion or insulin action or both. Diabetes has been reported to disrupt the functions and dynamics of mitochondria, which play a fundamental role in regulating metabolic pathways and are crucial to maintain appropriate energy balance. Similar to mitochondria, the functions and the abilities of stem cells are attenuated under diabetic condition in several tissues. In recent years, several studies have suggested that the regulation of mitochondria functions and dynamics is critical for the precise differentiation of stem cells. Importantly, physical exercise is very useful for preventing the diabetic alteration by improving the functions of both mitochondria and stem cells. In the present review, we provide an overview of the diabetic alterations of mitochondria and stem cells and the preventive effects of physical exercise on diabetes, focused on skeletal muscle and the nervous system. We propose physical exercise as a countermeasure for the dysfunction of mitochondria and stem cells in several target tissues under diabetes complication and to improve the physiological function of patients with diabetes, resulting in their quality of life being maintained.

## 1. Introduction

Diabetes mellitus (DM) is one of the most common metabolic diseases worldwide, and the number of patients with DM has continued to increase in recent years. Patients with DM exhibit hyperglycemia caused by an impairment in insulin secretion (type 1), insulin action (type 2), or both. Type 1 diabetes mellitus (T1DM), which accounts for less than 10% of diabetes cases, is characterized by an immune-mediated destruction of β cells in the pancreatic islets of Langerhans, leading to insulin deficiency [1]. It is well known that T1DM is developed in childhood and can lead to severe long-term complications including retinopathy, neuropathy, and nephropathy [2]. On the other hand, type 2 diabetes mellitus (T2DM), which accounts for less than 90% of diabetes cases, involves insulin resistance in peripheral tissues and increased levels of blood glucose due to overnutrition accompanied by deficient insulin secretion [3,4]. DM is often associated with the development of secondary complications in various organs, such as eyes, kidneys, heart, brain, and skeletal muscle [5].

The skeletal muscle is notably affected by DM. It has been shown that DM induces atrophy [6,7,8], fiber-type transition from oxidative to glycolytic [9,10], and impaired energy metabolism in skeletal muscle [11,12]. These alterations result in skeletal muscle dysfunction, such as muscle weakness and exercise intolerance [8,13]. Additionally, the central nervous system is critically influenced by DM. DM has been reported to induce pathological alterations in the nervous system that result in the onset of cognitive deficits and increase the risk for vascular complications in the brain [14]. Furthermore, DM is associated with vascular dementia, depression and Alzheimer’s disease (AD) [15,16,17,18,19]. These disorders may be caused by morphological changes, including white matter leukoaraiosis and hippocampal, cortical, and amygdala atrophies in the brains of the patients with DM [20,21].

Mitochondria and stem cell dysfunctions are among the multiple factors that can cause disturbances to the skeletal muscle and nervous system function in DM. Mitochondria play critical roles in regulating metabolic pathways and maintaining appropriate energy balance in tissues. DM is associated with reduced mitochondrial function, including decreased mitochondrial numbers [22] impaired lipid oxidation [23,24] and excessive production of reactive oxygen species (ROS) [25,26,27]. Additionally, the proliferation and differentiation of skeletal muscle stem cells, termed satellite cells, are attenuated in the diabetic skeletal muscle [28,29,30]. Moreover, the proliferative ability of neural stem cells (NSCs) is declined in the hippocampus of T1DM animal models [31,32]. The neurogenesis of NSCs is impaired in DM because of decreased expression of the transcription factor NeuroD1 [32,33]. These mitochondrial and stem cell dysfunctions may disrupt cell homeostasis, resulting in the disturbance of skeletal muscle and the brain function in DM.

It has been reported that the reduced myogenic potential of muscle stem cells is caused by mitochondrial dysfunction, including disturbed biogenesis [34] impaired dynamics [35] and high levels of ROS [36]. Similarly, precise mitochondrial function regulates the differentiation of NSCs in the adult hippocampus [37]. This crosstalk between mitochondria and stem cells may underlie the functional alterations in skeletal muscle and the nervous system under the diabetic condition.

The present review focuses on the diabetic alterations in mitochondrial and adult stem cell functions, and to an extent on the relationship between both, in skeletal muscle and the nervous system. Furthermore, based on the current body of knowledge, we propose physical exercise as a countermeasure for the diabetic complications in skeletal muscle and the brain.

## 2. Mitochondrial Dysfunction in Diabetes

### 2.1. Mitochondrial Content and Dynamics in Diabetic Muscle

**Figure 1:**
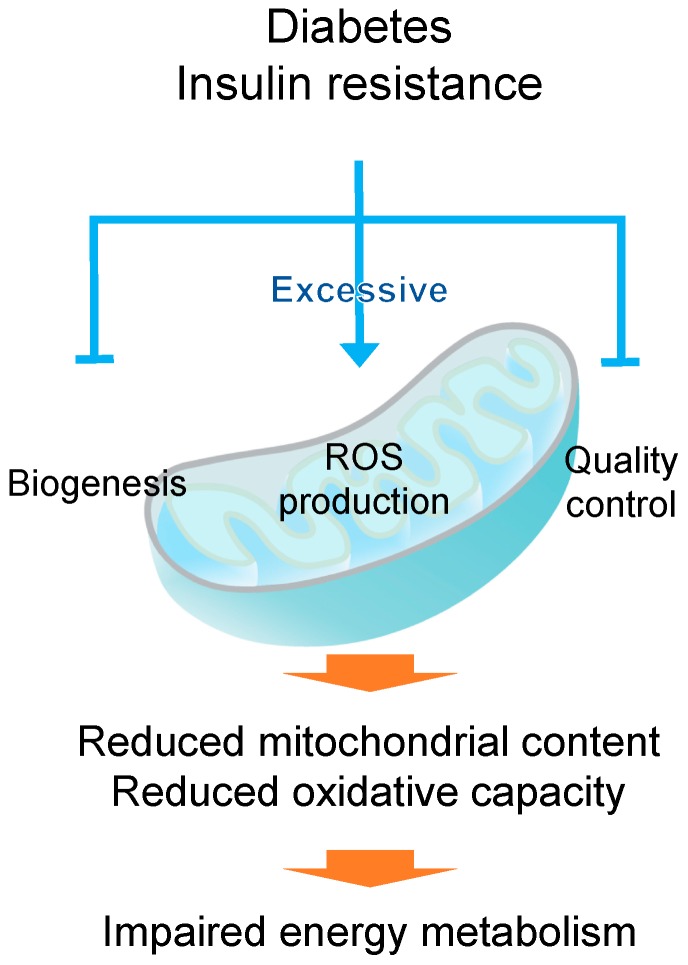
Schematic representation of mitochondrial dysfunction in diabetic skeletal muscle. Skeletal muscle contains a large volume of mitochondria that produce energy for biological activity. Diabetes mellitus induces mitochondrial dysfunction, including decreased biogenesis, impaired quality control (e.g., fusion, fission and mitophagy), and excessive ROS production in skeletal muscle, leading to the reduction in mitochondrial content and oxidative phosphorylation.

Skeletal muscle is highly plastic tissue that can adapt to the changes in energy status via changes in mitochondrial content. Previous studies examining the relationship between mitochondria and insulin resistance have reported that skeletal muscles of patients with T2DM exhibit reduced mitochondrial content [22,38,39]. Mitochondrial oxidative capacity is significantly lower in the skeletal muscle of insulin-resistant individuals than in that of healthy subjects, and this alteration results in increased fat accumulation in skeletal muscle [40]. Disturbed mitochondrial function has also been observed in cultured myocytes derived from skeletal muscle of patients with T2DM [41]. These findings indicate that mitochondrial content in skeletal muscle is reduced under the diabetic condition. Mitochondrial content is controlled by mitochondrial biogenesis (synthesis) [42,43], which is induced by various physiological, environmental, and pharmacological stimuli through promoting several regulators. Peroxisome proliferator-activated receptor γ coactivator-1α (PGC-1α) is a major regulator of mitochondrial biogenesis and function and modulates the expression of some genes associated with mitochondrial biogenesis interacting with nuclear respiratory factor 1 (NRF1); NRF1 promotes the expression of mitochondrial transcription factor A (TFAM), which is the final activator in the expression of mitochondrial DNA (mtDNA)-coded genes [44]. Muscle-specific PGC-1α-knockout mice have been reported to exhibit a oxidative-to-glycolytic muscle fiber-type shift and decreased expression of oxidative-related genes [45]. Accordingly, electrotransfection-mediated overexpression of PGC-1α in skeletal muscle resulted in increased PGC-1α protein levels, insulin sensitivity, and lipid oxidation [46]. Importantly, the expression of PGC-1α is reduced in the skeletal muscle of DM patients [22,23,47]; it is therefore supposed that diabetes-induced reduction in mitochondrial content is caused by downregulation of PGC-1α in skeletal muscle. PGC-1α binds and cooperates with its effectors including estrogen-related receptor α (ERRα) and peroxisome proliferator-activated receptor δ (PPARδ) [48]. The ability of PGC-1α to promote the expression of mitochondrial genes is severely impaired in the absence of ERRα [49]. However, the knockout of ERRα in skeletal muscle causes no phenotypic alteration in normal condition [50,51], suggesting that mitochondrial biogenesis induced by PGC-1α and ERRα is a transient process and that other transcription factors may regulate the basal-state levels of mitochondria. Additionally, PPARδ increases mitochondrial biogenesis and oxidative metabolism in skeletal muscle. Overexpression of PPARδ induced fiber-type transition from glycolytic to oxidative and increased mtDNA copy number and mitochondria-related proteins in skeletal muscle [52]. Conversely, muscle-specific deletion of PPARδ leaded to oxidative-to-glycolytic fiber-type shift with reduction in mitochondrial oxidative phosphorylation and fatty acid oxidation [53]. Obese T2DM patients exhibited decreased expression of *PPARδ* in skeletal muscle [54]. Taken together, diabetes leads to the impairment of mitochondrial biogenesis that may be caused by the downregulation of PGC-1α and/or PPARδ, resulting in reduced oxidative capacity in skeletal muscle (Figure 1).

Mitochondria are dynamic organelles that can flexibly adapt to the changes in cellular energy demands owing to continuous network remodeling through the process of fusion and fission [55]. Mitochondrial fusion in mammal cells is mediated by three large guanosine triphosphatases (GTPases) of the dynamin superfamily: mitofusin 1 (MFN1) and mitofusin 2 (MFN2), which are integral proteins in the outer membrane mediating outer-membrane fusion, and optic atrophy-1 (OPA1), which mediates inner membrane fusion [56]. Skeletal muscle-specific deletion of *MFN1* and *MFN2* causes severe mitochondrial dysfunction and loss of muscle mass, which are associated with increased mtDNA point mutations and mtDNA depletion [57]. Similarly, disruption of *OPA1* in mammal cells by RNA interference (RNAi) blocked mitochondrial fusion, leading to poor cell growth and mitochondrial dysfunctions, including decreased mitochondrial membrane potential and reduced cellular respiration [58]. This evidence indicates that mitochondrial fusion is essential for maintaining mitochondrial quality. Additionally, mitochondrial fission, which is mainly mediated by dynamin-related protein 1 (DRP1) and fission protein 1 (Fis1), plays an important role in mitochondrial quality control. DRP1 is a cytosol-located GTPase and is recruited by Fis1 to fission sites on the mitochondrial outer membrane to promote membrane division. Downregulation of *Drp1* by RNAi induced mitochondrial dysfunction in various cell lines [59,60], suggesting that mitochondrial fission is also required for maintaining mitochondrial quality and quantity. Several studies have indicated that DM influences the processes of mitochondrial fusion and fission. Bach et al., have shown that f MFN2 expression is lower in skeletal muscle of both non-diabetic obese subjects and T2DM patients than in that of healthy subjects [61]. Joseph et al., observed decreased expressions of the fusion proteins MFN2 and OPA1 in the skeletal muscle of T2DM patients [62]. Furthermore, high-fat diet-induced obese mice have exhibited the upregulation of fission proteins and downregulation of fusion proteins in skeletal muscle [63]. However, Pereira et al., have reported that OPA1-deficient young mice showed progressive mitochondrial dysfunction and loss of muscle mass, while they were tolerant to age- and diet-induced weight gain and insulin resistance through mechanisms that involve the activation of secretion of fibroblast growth factor 21 from skeletal muscle [64]. This study has suggested that blockage of mitochondrial fusion might increase the metabolic rate and improved whole-body insulin sensitivity. According to these results, it is supposed that DM probably disturbs mitochondrial dynamics in skeletal muscle (Figure 1), but detailed investigation is required to reveal the precise effects of DM on the processes of mitochondrial fusion and fission.

To maintain mitochondrial quality control, poorly functioning mitochondria are selectively degraded through mitophagy, which is the selective degradation of mitochondria by autophagy. Damaged mitochondria are taken up by autophagosomes, which fuse with lysosomes for catabolism of the mitochondria [65]. Mitophagy occurs in response to various alterations, such as changes in metabolic state, redox state, and nutrient availability. In mammals, one of the regulatory mechanisms of mitophagy is the PTEN-induced putative kinase 1 (PINK1)-PARKIN signaling pathway. PINK1 is a serine/threonine kinase that is imported into mitochondria and degraded by the mitochondrial rhomboid protease PARL in normal conditions. Mitochondrial depolarization and other stress conditions lead to the accumulation of PINK1 on the outer membrane, where PINK1 then phosphorylates the E3 ubiquitin ligase PARKIN. Activated PARKIN promotes the degradation of a number of mitochondrial proteins, including MFN1 and MFN2, and facilitates mitochondrial fragmentation, which enables mitophagy and prevents the re-fusion of poorly functioning mitochondria [66]. Alterations in mitophagy induced by DM have been insufficiently investigated. However, Scheele et al., have shown that *PINK1* expression is significantly lower in skeletal muscle of patients with T2DM than in control subjects [67]. This finding suggested that DM might inhibit appropriate mitophagy and thus, induce the accumulation of damaged mitochondria in skeletal muscle, leading to the disturbance of energy metabolism. Further investigation is required to deepen our understanding of alterations in mitochondrial quality control in the diabetic skeletal muscle.

### 2.2. Mitochondrial Reactive Oxygen Species (ROS) Production in Diabetic Muscle

Mitochondria are the principal organelles related to the production of ROS, which are generated as inevitable by-products of mitochondrial respiration. ROS include the superoxide anion radical (O_2_^•−^), hydroxyl radical (OH^•^), and hydrogen peroxide (H_2_O_2_). Excess ROS production in the absence of sufficient antioxidant capacity leads to lipid peroxidation and other oxidative stress, including the damages to the nuclear and mitochondrial DNA [68]. The relation between excess mitochondrial ROS production and skeletal muscle insulin resistance has been well established. By measuring total protein carbonylation and plasma H_2_O_2_ levels, Bonnard et al., found that oxidative stress in the skeletal muscle of T1DM model mice induced by treatment with streptozotocin (STZ) is higher than that of control mice. They also showed that obese mice fed a high-fat, high-sucrose diet display increased oxidative stress in the skeletal muscle. Moreover, T1DM mice and obese mice exhibited mitochondrial dysfunction, including decreased mtDNA copy number, increased number of disarrayed cristae, and reduced citrate synthase activity [69]. Anderson et al., reported increased mitochondrial H_2_O_2_ emission in obese human subjects as compared to healthy subjects, and the intake of a high-fat diet increased mitochondrial H_2_O_2_ production and oxidative stress in the skeletal muscle of healthy, insulin-sensitive subjects [70]. These findings suggest that DM promotes mitochondrial ROS production in skeletal muscle, leading to reduced mitochondrial content and function (Figure 1).

Approaches to oxidative stress suppression have been analyzed in previous studies. Hoehn et al., have demonstrated that genetic overexpression of manganese superoxide dismutase (MnSOD), an essential mitochondrial antioxidant enzyme detoxifying superoxide, and supplementation with mitochondrial O_2_^•−^-targeted antioxidant manganese (III) tetrakis (4-benzoic acid) porphyrin improve skeletal muscle insulin resistance in mice fed high-fat diets [71]. Furthermore, genetic overexpression of the mitochondria-targeted antioxidant human catalase and chronic intake of SS31, a small antioxidant peptide targeted to the mitochondrial inner membrane, resulted in the reduction of mitochondrial H_2_O_2_ production in skeletal muscle [70]. Thus, mitochondrial ROS production could be a causative factor of skeletal muscle insulin resistance and a key therapeutic target for the prevention of diabetes-induced mitochondrial dysfunction.

### 2.3. Alteration of Mitochondria in Neural Tissues

Diabetes complication induces mitochondrial dysfunction in neural tissues as well as skeletal muscle. Brain mitochondria of STZ-induced diabetic rats display decreased coenzyme Q9 [72], which suggests a disturbance of the antioxidant system in diabetic animals. Mastrocola et al., reported that brain mitochondria isolated from STZ-induced diabetic rats exhibits the decreased respiratory capacity and increased oxidative stress, which contributed to mitochondrial dysfunction by decreasing the activities of complex III, IV and V of the respiratory chain and ATP synthesis [73]. In addition, Cardoso et al., observed higher levels of malondialdehyde together with increased glutathione disulfide reductase and reduced MnSOD activities in hippocampal mitochondria isolated from STZ rats. Apart from T1DM, several reports indicate that T2DM or insulin resistance induces mitochondrial dysfunction in the brain. An age-related decline in respiratory chain efficiency and an uncoupling of oxidative phosphorylation systems have been observed in brain mitochondria of Goto-Kakizaki rats, a model of T2DM [74]. Carvalho et al., showed that brain mitochondria isolated from high-sucrose-induced T2DM mice functioned poorly, including lower respiration and membrane potential [75]. In their study, triple-transgenic AD model mice displayed phenotypes similar to T2DM mice [75]. Moreover, multiple studies have reported mitochondrial dysfunction in AD animal models [76,77,78]. These studies suggest that AD as a diabetic complication is caused by mitochondrial dysfunction due to insulin insensitivity.

## 3. Alteration of Stem Cell Function in Diabetes

### 3.1. Impairment of Muscle Stem Cell Function in Diabetes

Resident satellite cells in skeletal muscle contribute to the postnatal maintenance, growth, repair, and regeneration of skeletal muscle [79]. In healthy adult muscle, satellite cells are mitotically quiescent under normal physiological conditions but are activated in response to stimulation, such as muscle injury, to become myoblasts and proliferate extensively [80]. The majority of proliferated myoblasts then undergo myogenic differentiation to fuse to existing fibers or to generate new muscle fibers, whereas others return to a quiescent state to self-renew and maintain the stem cell pool [81]. Satellite cell-depleted mice exhibit poor regeneration after muscle injury [82], suggesting that satellite cells are essential for muscle regeneration.

Satellite cells demonstrate at least two states in skeletal muscle turnover: a quiescent state and an activated state. Both quiescent and activated satellite cells express the characteristic marker Pax7, whereas only activated satellite cells also express Myf5 and MyoD, which are key transcription factors for myogenic lineage progression and differentiation [83]. Although most Pax7^+^/MyoD^+^ satellite cells proliferate and then differentiate into a myogenic lineage through the downregulation of Pax7, others downregulate MyoD expression and withdraw from the cell cycle to return to a quiescent state [81,84]. MyoD transcription factor initiates the transcription of myogenin and other muscle-specific genes in differentiating myoblasts [85]. Thus, MyoD is regarded a master regulator of myogenesis by upregulating the transcription of skeletal muscle-specific genes.

Previous studies have shown that DM impairs satellite cell function. Satellite cells derived from STZ-induced diabetic mice are unable of myotube formation, resulting in poor regeneration after cardiotoxin-induced muscle injury [86]. Diabetic Akita mice also impaired muscle regeneration following injury caused by attenuated macrophage infiltration and satellite cell recruitment into degenerative muscle fibers [87]. Furthermore, the expression of the myogenic transcription factors MyoD and myogenin is reportedly decreased in gastrocnemius muscle from STZ-induced diabetic rats [28]. Recently, D’Souza et al., reported that skeletal muscles from human subjects with T1DM as well as diabetic animal models exhibit decreased satellite cell content [88]. Fujimaki et al., reported that decreases in total satellite cell content and the proportion of activated to total satellite cells in the STZ-induced diabetic skeletal muscle [30]. These studies suggest that T1DM leads to satellite cell dysfunction, including reductions in the number and myogenic capacity of cells, resulting in poor muscle regeneration following injury.

**Figure 2:**
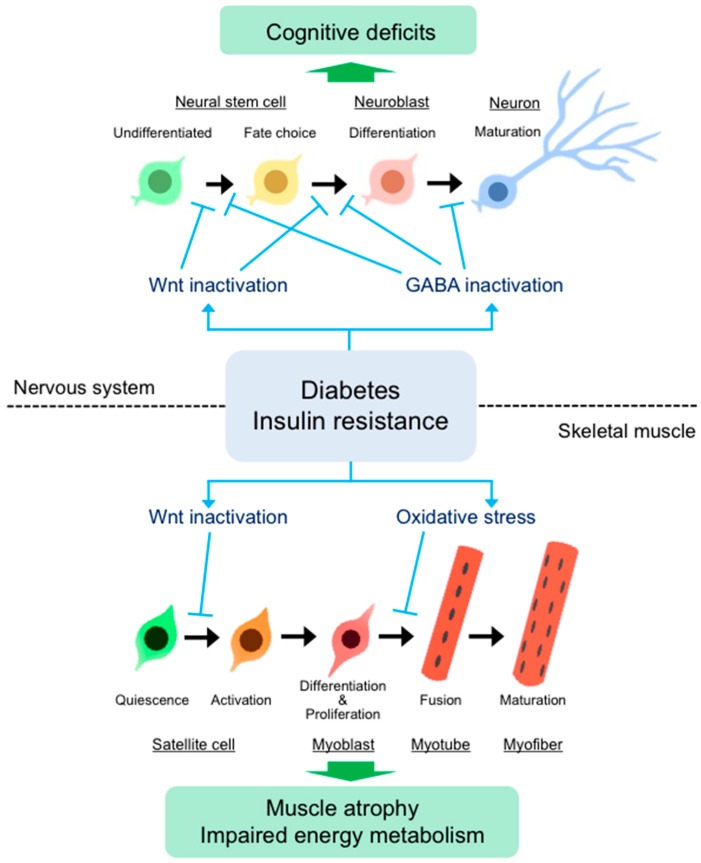
Schematic representation of regulation of stem cell differentiation in skeletal muscle and the nervous systems. Skeletal muscle stem cells, termed satellite cells, are mainly in a quiescent state, but activated in response to muscle injury or exercise. Activated satellite cells can proliferate, differentiate into myoblasts, and then fuse and mature into myofibers. Diabetes mellitus impairs satellite cell activation and differentiation via inactivation of Wnt signaling and/or excessive oxidative stress, resulting in muscle atrophy and reduced oxidative capacity in skeletal muscle. In adult brain, neural stem cells (NSCs) give rise to neuroblasts, which differentiate into mature neuron. The progression of NSCs to mature neuron is controlled by Wnt and γ-aminobutyric acid (GABA). Diabetes inhibits the activation of Wnt signaling and the expression of GABA transporters, resulting in disturbed neurogenesis, which may be associated with cognitive deficits.

The effects of T2DM on satellite cell function have been investigated using animal models. Nguyen et al., observed decreased proliferation of satellite cells and impaired muscle regeneration in transgenic *ob*/*ob* and *db*/*db* mice, which are common mouse models of T2DM [29]. Peterson et al., showed that obese Zucker rats display decreased satellite cell proliferation, with no change in the proportion of quiescent satellite cells [89]. This study also indicated declines in MyoD and myogenin protein levels in plantaris muscle from obese as compared to lean Zucker rats [89]. Additionally, there are some reports on the alteration of satellite cell function under the conditions of hyperglycemia and lipotoxicity, which are causes of T2DM. Hu et al., observed impaired muscle regeneration following cardiotoxin-induced injury in skeletal muscle from obese mice fed a high-fat diet for 8 months [90]. Similarly, shorter-term (3 months or 3 weeks) feeding of a high-fat diet also decreases the regenerative capacity through a decline of satellite cell numbers in skeletal muscle [91,92]. The effects of lipid overload on muscle regeneration have been investigated using transgenic mice overexpressing lipoprotein lipase, which converts triacylglycerol to free fatty acids and glycerol, in skeletal muscle. The transgenic mice displayed increased free fatty acid uptake in skeletal muscle and developed severe myopathy [93,94]. Ten days after muscle injury, cross-sectional areas of regenerating myofibers in the transgenic mice were smaller than those in wild-type control mice [94], indicating that lipid accumulation in skeletal muscle impairs regeneration. In addition, satellite cells derived from DM patients or model animals exhibit diabetic phenotypic characters, including increased expression of inflammatory cytokines [95] reduced lipid oxidation [41] disturbed glucose uptake [96] and insulin resistance [97]. According to these findings, impaired myogenic capacity of satellite cells may lead to disruption of muscle homeostasis, including atrophy and reduced energy metabolism, under diabetes complication (Figure 2).

The molecular mechanisms underlying satellite cell dysfunction induced by diabetes have been extensively investigated. Firstly, excess oxidative stress in the skeletal muscle is one of the candidate causes of the satellite cell dysfunction in DM. In both T1DM and T2DM, ROS production in the skeletal muscle is elevated as described in the preceding section. Studies have revealed that ROS in the skeletal muscle inhibits myogenic progression. Sandiford et al., showed that the overexpression of dual oxidase maturation factor 1 (DUOXA1), a member of the nicotinamide adenine dinucleotide phosphate oxidase (Nox) family that plays a critical role in ROS generation in a variety of cell types, leads to an increased H_2_O_2_ level, resulting in the inhibition of differentiation in the myoblast cell line C2C12, while a contrary phenotype was observed in a knockdown model of DUOXA1 [36]. Ardite et al., demonstrated that depletion of glutathione, an important and versatile antioxidant, in C2C12 cells impaired myogenic differentiation as indicated by lower creatine kinase activity, expression of MyoD and myosin heavy chain, and myotube formation, through the upregulation of NF-κB [98]. Guttridge et al., reported that NF-κB inhibits myogenic differentiation via increased cyclin D1 expression and cell proliferation, and decreased MyoD expression [99,100]. On the other hand, some investigators have argued that NF-κB is essential for myogenic progression. While NF-κB may regulate myogenesis both positively and negatively, further investigations are required for an appropriate understanding of its function in satellite cell differentiation. Additionally, ROS induces decreased expression of PGC-1α and mitochondrial disruption [101,102], while proper mitochondrial function is essential for muscle regeneration [34,103], indicating that mitochondrial function is closely connected with myogenic progression. Together, these findings suggest that oxidative stress decreases the myogenic potential of satellite cells in diabetic muscle.

Secondly, diabetes-induced dysfunction of satellite cells is caused by the alteration of Notch and Wnt signaling. Notch signaling regulates cell fate and proliferation in satellite cells. The binding of notch receptors to their δ/jagged, serrate, or lag2 (DSL) ligands releases the Notch intracellular domain [104]. This domain then associates with recombining binding protein-Jĸ, which is a key transducer of notch signaling [105,106], and then translocates into the nucleus to promote Hes and Hey transcription [84]. Notch signaling blocks differentiation and contributes to maintaining the quiescence of satellite cells [84]. D’Souza et al., showed that notch activity in satellite cells derived from wild-type mice is downregulated upon conversion from quiescent to activated state, while in Akita diabetic mice, it remains activated under this condition. The authors discussed that the hyperactivation of the notch signaling pathway impaired the myogenic capacity of satellite cells in T1DM [88]. On the other hand, a previous study demonstrated that the downregulation of Wnt signaling activity leads to impaired myogenic differentiation of satellite cells [30]. Wnt is a secreted extracellular ligand that binds to Frizzled receptor located in the plasma membrane [107,108], and stabilizes β-catenin, which forms a complex with the T-cell factor (TCF)/leukocyte enhancer factor (LEF) that translocates into the nucleus to activate the transcription of target genes [109,110]. Wnt signaling regulates myogenesis via the modulation of *MyoD* expression [111]. Fujimaki et al., reported that STZ-induced diabetes inhibits satellite cell activation induced by decreased Wnt signaling activities, including the gene expressions of Wnt ligands and β-catenin accumulation [30]. Although the alteration of notch and Wnt signaling is associated with diabetes-induced dysfunction of satellite cells, further investigation is required for a clear understanding of the molecular mechanisms underlying impaired satellite cell function in DM.

### 3.2. Impairment of Neural Stem Cell Function in Diabetes

Neurogenesis in the adult mammalian brain is a multistep process, including proliferation of neural progenitor cells, fate determination, migration, neuronal maturation, and functional integration of newborn cells into the existing neuronal circuitry [112]. NSCs are primarily located in two distinct regions of the brain: the subventricular zone (SVZ) of the lateral ventricles and the subgranular zone of the hippocampal dentate gyrus (DG). In the SVZ, adult NSCs give rise to neuroblasts, which migrate into the olfactory bulb (OB) through the rostral migratory stream and then differentiate into mature local interneurons. In the DG, proliferating neuroblasts become immature neurons and project their axons into the CA3 region of the hippocampus. These immature neurons eventually differentiate into mature neurons and are integrated into the existing hippocampal circuitry as functional granule cells. Recent studies have shown that newly formed neurons are incorporated into the functional networks of both the OB and the DG, suggesting that adult neurogenesis notably affects brain functions associated with learning, memory processing, and odor discrimination [113,114,115,116,117].

Several studies have demonstrated that Wnt signaling regulates adult neurogenesis. For example, Wnt3 is strongly expressed in astrocytes of neurogenic niche and NSCs expressed the major components of the Wnt signaling pathway [118,119]. In coculture study of NSCs with hippocampal astrocytes, astrocyte-derived Wnts activate neuroblast proliferation and neuronal differentiation [118]. Interestingly, NeuroD1, a key transcription factor for neurogenic lineage progression and one of the major targets of Wnt signaling, is selectively expressed in dividing neural progenitors and immature granule neurons, but not in Sox2-expressing NSCs. Kuwabara et al., reported that the NeuroD1 promoter can bind to Sox2 and the TCF/LEF complex. Their study has suggested that NeuroD1 transcription is activated by Wnt signaling in NSCs during neurogenesis, while it is suppressed by Sox2 when neurogenesis is inhibited [120]. Furthermore, using NeuroD1 conditional knockout mice, Gao et al., found that NeuroD1 is required for adult neurogenesis both in vivo and in vitro [121]. Our previous study also showed that NeuroD1 directly activates insulin gene expression in NSCs from adult hippocampus and OB, resulting in the induction of neuronal differentiation [122]. According to these evidences, the Wnt-NeuroD1 axis plays an essential role in neurogenesis in the adult hippocampus and OB.

Accumulating evidence has demonstrated that adult neurogenesis in the brain is disturbed by DM. STZ-induced diabetes consistently decreases hippocampal cell proliferation in rodents [31,32,123,124,125,126,127]. Decreased immature neurons were observed in STZ-induced diabetic animals through Bromodeoxyuridine (BrdU) incorporation analysis [31,32], indicating that neuronal differentiation is inhibited by DM. In addition, the proportion of mature neurons in STZ-induced diabetic rats has been shown to be either decreased [32] or unchanged [125]. Similar to STZ-induced diabetic animals, non-obese diabetic (NOD) mice, which are another model of T1DM developed by the autoimmune destruction of pancreatic β cells [128], exhibit decreased hippocampal cell proliferation [129,130] and disturbed neuronal differentiation [130]. Taken together, T1DM models consistently show decreased hippocampal cell proliferation and survival, and in some studies, these models were also exhibited disturbed neuronal differentiation. Hippocampal neurogenesis has been studied in various animal models of T2DM, including *db*/*db* mice and Zucker diabetic fatty (ZDF) rats, which are leptin-receptor-deficient and are used as models of obesity complicated by diabetes. ZDF rats display decreased hippocampal cell proliferation and neuronal differentiation as measured by Ki67 or doublecortin immunoreactivity [131]. Similarly, *db*/*db* mice show reduced hippocampal cell proliferation when compared to control mice [132]. These studies suggest that adult neurogenesis is severely impaired in T2DM (Figure 2).

Our previous study indicated that NeuroD1 and insulin expression is decreased in NSCs derived from the hippocampus and OB of STZ-induced diabetic rats, which exhibit loss of neurogenic potential of NSCs [33]. Recently, we showed that STZ-induced T1DM induces disturbed neurogenic differentiation of NSCs and reduced expression of Wnt3 and NeuroD1 in the OB, resulting in several behavioral deficits, including impaired odor discrimination, cognitive dysfunction, and increased anxiety [133,134]. The inhibition of Wnt signaling in the DG of adult rats reportedly impairs spatial memory and object recognition [134]. These results suggest that diabetes-induced cognitive deficits may be attributed to the downregulation of Wnt signaling. Additionally, we have provided evidence that DM alters neurotransmitter systems, such as γ-aminobutyric acid (GABA) and glutamate transporters. GABA and glutamate are the principal inhibitory and excitatory neurotransmitters, respectively, in mammalian central nervous systems, and their transporters modulate adult neurogenesis [135,136,137,138,139]. The expressions of GABA transporters (GATs), excitatory amino acid transporters, and vesicular glutamate transporter is decreased in the OB of STZ-induced diabetic as compared to healthy rats [133]. Furthermore, GAT1 inhibition disturbs Wnt3-induced neuronal differentiation of NSCs in vitro [133]. According to this study, the regulation of local GABA and glutamate neurotransmitter levels is important for the maintenance of adult neurogenesis and can be a therapeutic target to prevent neuronal dysfunction induced by DM that results in cognitive deficits (Figure 2).

## 4. Mitochondrial Function in Stem Cell Differentiation

There has been various research on the crosstalk between mitochondria located in mature muscle fibers and satellite cell function. Wagatsuma et al., demonstrated that the activity of citrate synthase dramatically increased soon after muscle injury when the myoblast began to differentiate into myotubes with increased expression of mitochondrial biogenesis-related genes, *NRF1*, *NRF2*, and *TFAM*, and myogenic regulatory factors, including *MyoD* and *myogenin* [34]. The authors also found that pharmacological blocking the synthesis of mitochondrial protein using chloramphenicol induces deficient regeneration and muscle fibrosis [34]. LaBarge et al., reported that muscle fiber-specific ERRα knockout mice exhibits impaired muscle regeneration with reduced mitochondrial content and citrate synthase activity compared to wild-type mice [51]. Furthermore, broad-acting autophagy inhibitor disturbed functional muscle regeneration and mitochondrial remodeling after injury [35], indicating that appropriate degradation of poorly functioning mitochondria by mitophagy is important for muscle regeneration. Additionally, oxidative stress decreases myogenic potential of satellite cells as described in the preceding section [36,98]. These findings suggest that mitochondrial function may be critical for precise differentiation of satellite cells in adult skeletal muscle.

It has also been reported that mitochondria in stem cells regulates their differentiation. As for skeletal muscle stem cells, Kim et al., have shown that C2C12 myoblasts treated with the mitochondrial division inhibitor *mdivi-1*, a specific inhibitor of DRP1 GTPase activity, display extensive formation of elongated mitochondria along with increased apoptosis. *Mdivi-1*-treated C2C12 myotubes showed dose-dependent reduction in mtDNA copy number, mitochondrial mass, and membrane potential, indicating disturbed mitochondrial biogenesis during myogenic differentiation. Furthermore, *mdivi-1* treatment significantly inhibited myotube formation in both C2C12 and primary myoblasts, suggesting that DRP1-dependent mitochondrial division is required for successful myogenic differentiation [140]. In the case of NSCs, Rharass et al., have demonstrated that mitochondrial ROS produced from human neural progenitors by growth factor depletion activate Wnt/β-catenin signaling, leading to neuronal differentiation. The authors also found that low levels of ROS suppress the activation of Wnt/β-catenin signaling owing to blockade of the Wnt effector Dishevelled, which resulted in notable impairment of neuronal differentiation [141]. The authors suggested that mitochondrial ROS may contribute to the precise adult neurogenesis. Beckervordersandforth et al., found that *TFAM*-deficient NSCs display a severe defect in neurogenic lineage progression [142]. The decreased neurogenic capacity is exhibited in *PINK1*-deleted NSCs [143]. Taken together, diabetes-induced inhibition of stem cell differentiation may occur through disturbed function of mitochondria. To verify this hypothesis, detailed studies on whether or not diabetes can induce mitochondrial dysfunction in stem cells are needed.

## 5. Preventive Effects of Physical Exercise on Diabetic Alterations

### 5.1. Response of Mitochondria in Diabetic Muscle to Exercise

Physical exercise can change mitochondrial content, shape, and function in skeletal muscle [144]. Firstly, mitochondrial biogenesis in skeletal muscle is enhanced by exercise. Endurance exercise stimulates mitochondrial biogenesis [145,146], which has been largely attributed to the cumulative effects of each bout of exercise sustained training [147,148]. PGC-1α expression responds to physical exercise as the muscle adapts to metabolic demands, which leads to mitochondrial biogenesis [43,149]. Both acute exercise and long-term training reportedly increase the expression of PGC-1α protein in the skeletal muscle [150,151]. However, it remains unclear whether exercise- and training-induced promotion of mitochondrial biogenesis requires for functioning PGC-1α. A study using PGC-1α-knockout mice showed that PGC-1α is not essential for the training-induced increase in the expressions of mitochondrial proteins, such as ALSA1, Cox1, and cytochrome C [152]. Additionally, muscle-specific PGC-1α-knockout mice exhibit exercise capacity and exercise-induced mitochondrial biogenesis similar to that of their wild-type littermates [153]. Therefore, other factors likely regulate the mitochondrial biogenesis accompanying with exercise and training. Exercise-induced mitochondrial biogenesis occurs along with an increase in mtDNA copy number. Interestingly, protein expression of TFAM is elevated in the skeletal muscle of both animals [154,155,156] and human [157] following endurance exercise. This upregulation of TFAM has been also observed in in vitro studies using contractile models of myotubes [158,159]. Based on these evidences, it is supposed that TFAM regulates mtDNA transcription and contributes to the increased expression of mitochondrially encoded genes resulting in the promotion of mitochondrial biogenesis in response to physical exercise [43]. Exercise-induced mitochondrial biogenesis has been observed in diabetic as well as healthy skeletal muscle. Patients with T2DM have shown to respond to endurance training with increases in insulin sensitivity and mitochondrial protein contents in the skeletal muscle [24,160]. Other styles of exercise training, including strength and concurrent training, have been also reported to increase the mitochondrial content in skeletal muscle of patients with T2DM [161,162]. Furthermore, a study mimicking exercise stimulation via electrotransfection of PGC-1α into rat skeletal muscle indicated increased PGC-1α protein content, mtDNA copy number, and mitochondrial enzyme activities, together with improvement of insulin sensitivity in the skeletal muscle [46]. These results suggest that exercise-induced upregulation of PGC-1α has beneficial effects on mitochondrial function in the diabetic muscle. Physical exercise also activates AMP-activated protein kinase (AMPK), which is activated under the condition of decreased ATP/AMP ratio such as exercise [163] and caloric restriction [164]. Importantly, AMPK phosphorylates and activates PGC-1α to promote the expression of mitochondria-related genes [165,166]. Acute exercise induced AMPK activation in skeletal muscle of T2DM patients [167], suggesting that the activation of the AMPK–PGC-1α axis may contribute to exercise-induced mitochondrial biogenesis. Additionally, exercise promotes the expression of PPARδ in skeletal muscle. Luquet et al., reported that 6 weeks of moderate exercise induces the accumulation of PPARδ protein in skeletal muscle [168]. Four months of low-intensity exercise training upregulated the expression of PPARδ with improvement of insulin sensitivity in skeletal muscle of T2DM patients [169]. Recently, specific PPARδ agonists have been reported to be effective to improve metabolic syndrome. Specific PPARδ agonist GW501516 reduced adiposity and improved insulin sensitivity in skeletal muscle of *db*/*db* mice or obese mice fed a high-fat diet [170,171]. Because PPARδ agonist and AMPK agonist upregulated metabolic genes and enhanced endurance capacity without exercise [172], they can be exercise mimetics. These findings suggest that AMPK–PPARδ pathway may be a therapeutic target for treatment of DM. Altogether, physical exercise can be an effective measure for DM patients to increase mitochondrial content with enhanced oxidative capacity in skeletal muscle.

Secondly, physical exercise also contributes to mitochondrial quality control in skeletal muscle. Kitaoka et al., demonstrated that the expressions of the mitochondrial fusion proteins MFN1, MFN2, and OPA1 is increased in skeletal muscle following electrical stimulation-induced resistance exercise training [173]. Similarly, swimming endurance training induces increased protein levels of mitochondrial fusion genes [174], suggesting that exercise training can accelerate mitochondrial fusion. Additionally, the expression of Fis1 and the activation of DRP1 are elevated after or during acute exercise [175], indicating that physical exercise increases mitochondrial fission in the skeletal muscle. Furthermore, 6 weeks of exercise training increased *PINK1* mRNA expression in human skeletal muscle [176], and PARKIN protein was decreased in the fasted state following acute exercise, which suggests that exercise promotes mitophagy [177]. Although these studies suggest that exercise training contributes to mitochondrial quality control, further investigation is needed to verify the effects of exercise on the improvement of mitochondrial quality in the diabetic muscle.

Thirdly, exercise training and muscle contraction lead to increased ROS production and oxidative stress [178,179]. Although excessive ROS can damage contractile proteins and organelles in the skeletal muscle [180], moderate oxidative stress plays important roles in muscle signaling and maintaining muscle homeostasis [181,182]. Indeed, healthy wild-type mice treated with antioxidants exhibited mitochondrial dysfunction leading to exercise intolerance [183]. Thus, balanced ROS production is critical for maintaining cellular function. In a previous study, 10 weeks of aerobic training suppressed excess mitochondrial H_2_O_2_ production in skeletal muscle of the patients with T2DM, leading to improved mitochondrial respiration [184]. Oxidative stress during exercise maintains mitochondrial fitness [185,186] and induces molecular regulators of insulin sensitivity and antioxidant defense [187]. Taken together, physical exercise may contribute to the inhibition of excessive ROS production in the skeletal muscle under diabetes complication. Future research needs clinical studies because there is still a gap between basic research and clinical application [188].

### 5.2. Effects of Exercise on Muscle Stem Cell Function

A number of studies have shown that physical exercise has positive effects on satellite cells. Satellite cell number have been reported to increase in animal models after acute or chronic exercise [189,190]. This increment in the satellite cell number is also observed in human skeletal muscle. The long-term effect of exercise on satellite cell number is apparent in the skeletal muscle of well-trained power lifters, who have 70% more satellite cells than the control subjects [191]. The increased number of satellite cells after exercise training gradually reduces during detraining [192], suggesting that a continuation of exercise is required for maintaining an abundant pool of satellite cells in skeletal muscle. Effective methods of exercise for increasing or maintaining the pool of satellite cells are still investigated [193,194]. Recently, Fujimaki et al., showed that the number of satellite cells in diabetic mice that performed treadmill running for 4 weeks was larger than that in control mice [30]. This suggests that exercise contributes to recovery of satellite cell numbers in DM.

Physical exercise is useful to increase not only satellite cell number but also satellite cell function. Four weeks of voluntary wheel running led to upregulation of Wnt signaling, which regulates to the activation and myogenic progression of satellite cells in skeletal muscle, in diabetic mice [111]. Consistent with this study, Aschenbach et al., demonstrated that acute treadmill running upregulates β-catenin through GSK-3β inactivation [195]. Moreover, functional overload, a model of resistance training that leads to muscle hypertrophy, induced β-catenin activation in the plantaris muscle [196]. Using chromatin immunoprecipitation assays, Fujimaki et al., demonstrated that the exercise-induced upregulation of Wnt signaling directly modulates the chromatin structures of the *Myf5* and *MyoD* and facilitates their transcription in adult satellite cells, resulting in increased mRNA expression of these genes and the satellite cell activation [111]. Furthermore, satellite cells derived from hypertrophic muscle induced by functional overload had improved their proliferative ability and myogenic capacity [197]. Fujimaki et al., further showed that the proportion of activated to total satellite cells is decreased in STZ-induced diabetic muscle as compared to healthy muscle. However, running exercise increased the proportion of activated satellite cells in diabetic muscle as well as healthy muscle through the upregulation of Wnt signaling [30], indicating that exercise inhibits the disturbance of satellite cell activation by DM. These results suggest that exercise can be a countermeasure for the dysfunction of satellite cells in the skeletal muscle under diabetes complication.

### 5.3. Effects of Exercise on Adult Neurogenesis

Physical exercise positively affects adult neurogenesis as well as myogenesis. Van Praag et al., demonstrated that voluntary running exercise promotes cell proliferation, cell survival, and neurogenesis in the DG of adult mice [198]. Exercise-induced increases in neurogenesis in the DG of the hippocampus have been reported in young, adult, and aged animals [199,200,201,202,203,204]. Furthermore, physical exercise improves the cognitive functions in aged mice and humans. These results suggest that exercise-enhanced adult neurogenesis leads to the improvement of cognitive functions. There are few reports on the preventive effects of exercise on neuronal dysfunctions in diabetes. Although physical exercise did not particularly affect body weight and blood glucose in STZ-induced diabetic rats, the reduction of hippocampal cell proliferation by DM was inhibited by exercise [205]. In addition, forced treadmill running increased hippocampal cell proliferation and differentiation, which are disturbed in the hippocampus of ZDF rats [206,207]. Physical exercise recovers cognitive deficits in STZ-induced diabetes as indicated by novel object recognition task, step-down avoidance task, and 8-arm radial maze testing [208,209]. These studies provide evidence that physical exercise improves adult neurogenesis and cognitive deficits in diabetes, suggesting that exercise can contribute to the recovery of diabetic complications in the central nervous system.

Recently, the molecular mechanisms underlying the exercise-induced promotion of cell proliferation and adult neurogenesis in the hippocampus have been gradually revealed. Exercise modulates the expressions of Wnt signaling-related genes in the hippocampus [210]. We previously demonstrated that running exercise induces enhanced expression of Wnt3 in the astrocytes of the DG and increases the population of Wnt3-expressing cells in both young and aged mice [211]. Furthermore, Mir et al., reported that exercise-induced neurogenesis depends on the novel RIT1/Akt/Sox2 cascade in the hippocampus. The author showed that gene deletion of *RIT1*, a Ras-related GTPase that is expressed throughout the central nervous system, blocks both exercise-induced and Insulin-like growth factor-1 (IGF-1)-dependent cell proliferation and differentiation of NSCs in the hippocampus. The study also demonstrated that IGF-1-dependent activation of Sox2, which is involved in the maintenance and proliferation of NSCs, is regulated by RIT1-Akt signaling and this cascade contributes to the proliferation and differentiation of NSCs in the hippocampal DG [212]. Additionally, vascular endothelial growth factor secreted by skeletal muscle has been suggested to regulate hippocampal blood flow and neurogenesis [213]. Although physical exercise may promote neurogenesis in diabetes via these regulators, further investigation is required for a detailed understanding of the mechanism of the preventive effects of exercise on neuronal dysfunction in DM.

## 6. Conclusions

The present review described diabetes-related alterations of mitochondria and stem cells in the skeletal muscle and central nervous system. In both skeletal muscle and the brain, diabetes induces mitochondrial dysfunction, including decreased mitochondrial respiration, reduced oxidative phosphorylation, and increased oxidative stress. Diabetes also interferes with stem cell function. The number and differentiation ability of satellite cells are decreased in diabetic skeletal muscle, which may be induced by the excess ROS production and/or inactivation of the notch and Wnt signaling pathways. Adult neurogenesis is also disturbed in the brain in case of diabetic complication via the downregulation of Wnt signaling. Because some reports indicate that precise differentiation of muscle and neural stem cells is controlled by mitochondrial function, the disturbances of myogenesis and neurogenesis may be induced by mitochondrial dysfunction in diabetes. Importantly, exercise is very useful for preventing/improving diabetic alterations in the skeletal muscle and central nervous system. Physical exercise leads to increased mitochondrial content and oxidative capacity in both healthy and diabetic muscle, and can block excessive ROS production induced by diabetes. Additionally, lineage progression of satellite cells and NSCs is accelerated by physical exercise through the upregulation of Wnt signaling. Although more investigation is required for a thorough understanding of diabetes-related alterations and biological mechanisms in various tissues, the current literature as presented in this review clearly suggests physical exercise to be a valuable measure for DM patients to prevent diabetic complications as well as to maintain or improve their quality of life.
